# Construction of NIR Light Controlled Micelles with Photothermal Conversion Property: Poly(poly(ethylene glycol)methyl ether methacrylate) (PPEGMA) as Hydrophilic Block and Ketocyanine Dye as NIR Photothermal Conversion Agent

**DOI:** 10.3390/polym12051181

**Published:** 2020-05-21

**Authors:** Lan Yao, Haihui Li, Kai Tu, Lifen Zhang, Zhenping Cheng, Xiulin Zhu

**Affiliations:** Suzhou Key Laboratory of Macromolecular Design and Precision Synthesis, State and Local Joint Engineering Laboratory for Novel Functional Polymeric Materials, College of Chemistry, Chemical Engineering and Materials Science, Soochow University, Suzhou 215123, China; 20174209022@stu.suda.edu.cn (L.Y.); harvey1119@163.com (H.L.); sudatukai@163.com (K.T.); xlzhu@suda.edu.cn (X.Z.)

**Keywords:** amphiphilic block copolymer, self-assembly, NIR photothermal conversion, reversible addition-fragmentation chain transfer (RAFT) polymerization

## Abstract

Polymeric nanomaterials made from amphiphilic block copolymers are increasingly used in the treatment of tumor tissues. In this work, we firstly synthesized the amphiphilic block copolymer PBnMA-*b*-P(BAPMA-*co*-PEGMA) via reversible addition-fragmentation chain transfer (RAFT) polymerization using benzyl methacrylate (BnMA), poly (ethylene glycol) methyl ether methacrylate (PEGMA), and 3-((*tert*-butoxycarbonyl)amino)propyl methacrylate (BAPMA) as the monomers. Subsequently, PBnMA-*b*-P(APMA-*co*-PEGMA)@NIR 800 with photothermal conversion property was obtained by deprotection of the *tert*-butoxycarbonyl (BOC) groups of PBAPMA chains with trifluoroacetic acid (TFA) and post-modification with carboxyl functionalized ketocyanine dye (NIR 800), and it could self-assemble into micelles in CH_3_OH/water mixed solvent. The NIR photothermal conversion property of the post-modified micelles were investigated. Under irradiation with NIR light (*λ*_max_ = 810 nm, 0.028 W/cm^2^) for 1 h, the temperature of the modified micelles aqueous solution increased to 53 °C from 20 °C, which showed the excellent NIR photothermal conversion property.

## 1. Introduction

As a research hotspot in recent years, the application of nanomaterials in biomedicine [1,2,3], information transmission [4,5,6], and other fields is becoming more and more extensive. Among various types of nanomaterials, the nanomaterials formed by self-assembly of amphiphilic block copolymers show brilliant performance in tumor treatments. Amphiphilic block copolymers can spontaneously self-assemble into nano-aggregates of various morphologies due to the different solubility of the hydrophilic chains and lipophilic chains [7,8,9]. poly (ethylene glycol) methyl ether methacrylate (PEGMA) is a highly hydrophilic material with excellent biocompatibility and is usually used as hydrophilic monomer in the design of amphiphilic block copolymers [10,11,12]. It is suitable for a variety of polymerization methods, such as RAFT polymerization [13,14,15], atom transfer radical polymerization (ATRP) [16,17,18], etc. During the self-assembly of PEGMA based amphiphilic block copolymer, the PEG chains of the micelle hydrophilic shell are fully stretched in water to prevent entanglement and aggregation between the chains, and the stability of micelles is greatly increased.

The structure of the amphiphilic block copolymer can be easily controlled. We could obtain copolymers with different monomers, molecular weights, and ratios of hydrophilic and hydrophobic chains [19,20,21,22]. Through the design of the structure, we could get aggregates with a size of 10–1000 nm via self-assembly. We all know that micelles with a size of 10–100 nm can take advantage of the enhanced permeability and retention (EPR) [23,24,25] effect of tumors to spontaneously enter the tumor tissues and plays a role in tumor targeted therapy. From this perspective, amphiphilic block copolymers are extremely promising in tumor targeted therapy due to their structural design.

According to the fact that certain tumor cells are more sensitive to heat, and photothermal materials can convert light energy into heat energy, nanomaterials with photothermal conversion property are transport to the tumor issue to kill tumors under light in photothermal therapy (PTT). Because of the strong penetrating power of NIR light, nanomaterials with NIR photothermal conversion property become the most widely used materials in PTT [26,27]. In order to enhance the tumor treatment effect, the combination of chemotherapy and PTT emerges [28,29,30]. However, inorganic NIR photothermal materials are not ideal photothermal agents due to their poor biocompatibility in the body, and their difficulty to clear away. In recent years, a lot of work has looked at micelles loading with photothermal materials, but the stability and photothermal conversion efficiency of these micelles are urgent problems to be solved.

Usually, dyes with small molecules—such as indocyanine green (ICG) [31,32], boron dipyrromethene dyes (BODIPY) [33,34,35], and cyanine dyes [36,37,38]—are loaded in the micelles for the use of PTT. Due to their excellent optical properties, high absorption strength, and good photothermal stability, the cyanine dyes have wide applications in many fields including nonlinear optics, organic photoconductors, and photodynamic therapy [39,40,41,42]. Squarylium dyes and ketocyanine dyes both belong to cyanine dyes. Compared with squarylium dyes, the ketocyanine dyes have stronger electron withdrawing ability and its maximum absorption wavelength (*λ*_max_) could be changed by adjusting the chromophore structure. Nowadays, ketocyanine dyes with the maximum absorption wavelength between 750–1100 nm have been synthesized through various synthetic methods [43,44,45]. Ketocyanine dyes are often used in the preparation of functional materials. Foley et al. synthesized NIR dye 2,5-bis[(4-carboxylic-piperidylamino)thiophenyl]-croconium with the *λ*_max_ at 800 nm. By reacting with KHCO_3_, a water-soluble salt containing this NIR dye was obtained. Then polymeric film consisting of this salt was synthesized through a water-based coating process and it can be used as NIR blocking plastic filters [46]. Liu Li et al. designed and synthesized a new four-armed NIR absorbing cross-linker, 2,5-bis[(1-dec-9-enyl-undec-10-neyl-4-carboxylate-piperidyl-amino)-thiophenyl] croconium. It was used in the acyclic diene metathesis polymerization of liquid crystal monomer 4-undec-10-enyloxy-benzoic acid 4-dec-9-enyloxy-phenyl ester. As a result, the mainchain LCE soft actuator material was successfully prepared. Under NIR light (808 nm) irradiation, the temperature of this material can raise from 18 °C to 260 °C in 8 s due to the extraordinary photothermal conversion property of NIR absorbing cross-linker [47]. The materials consisting ketocyanine structure mentioned above present significant NIR photothermal conversion performance; however, there is not much research about ketocyanine dyes which are used in tumor treatment.

**Scheme 1:**
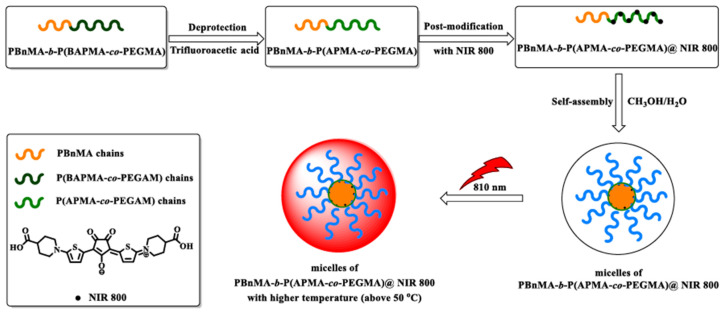
Schematic of deprotection, post-modification, micellization, and NIR light triggered temperature rising process of PBnMA-*b*-P(BAPMA-*co*-PEGMA).

Based on the efficient NIR photothermal conversion property of ketocyanine dyes, in this study, we introduce the controlled synthesis, self-assembly, and NIR photothermal conversion property of an amphiphilic block copolymer containing ketocyanine dyes. The block copolymer PBnMA-*b*-P(BAPMA-*co*-PEGMA) was synthesized via RAFT polymerization. It can self-assemble in the mixed solvent of CH_3_OH/H_2_O. We designed the degree of polymerization of BnMA and PEGMA to adjust the size of micelles. Under the action of TFA, we obtained the deprotected block copolymers, which could react with NIR 800 and resulting the post-modified block copolymer PBnMA-*b*-P(APMA-*co*-PEGMA)@NIR 800. The NIR photothermal conversion property of NIR 800 and post-modified micelles were evaluated. According to the results, both the solution of NIR 800 and post-modified micelles have excellent NIR photothermal conversion performance. The schematic deprotection, post-modification, micellization, and NIR light triggered temperature rising process of PBnMA-*b*-P(BAPMA-*co*-PEGMA) in aqueous solution are shown in Scheme 1. In this work, we have successfully prepared micelles that have excellent NIR photothermal conversion performance, which have the potential to be used in both tumor targeted therapy and photothermal therapy.

## 2. Experimental Section

### 2.1. Materials

Benzyl methacrylate (BnMA, 98%) was purchased from Aladdin Reagent Co. Ltd. (Shanghai, China). and purified by passing through a neutral alumina column. Poly(ethylene glycol) methyl ether methacrylate (PEGMA, 98%, *M*_n_ = 500 g/mol) was purchased from Sigma-Aldrich (Shanghai, China) trading Co., Ltd. and purified by passing through a neutral alumina column. 2,2′-azobis(2-methylpropionitrile) (AIBN, 98%, Adamas) was recrystallized twice in ethanol before use. Thiophenethiol (99%, TCI), methyl 4-piperidinecarboxylate (98%, Energy Chemical, Shanghai, China), croconic acid (AR, J&K, Shanghai, China), methacryloyl chloride (99%, TCI, Tokyo, Japan), pyrene (AR, Adamas, Shanghai, China), 3-(BOC-amino)-1-propanol (97%, Energy Chemical, Shanghai, China), trifluoroacetic acid (TFA, 99%, Energy Chemical), and acetic acid (99%, Aladdin, Shanghai, China) were used as received. All other chemicals were purchased from Chinasun Specialty Products Co. Ltd. (Suzhou, China). and used without purification.

### 2.2. Synthesis of BAPMA

**Scheme 2:**
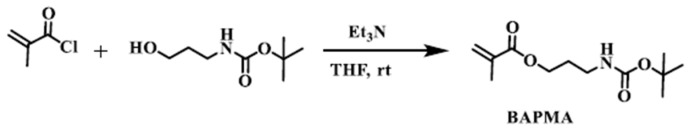
Synthetic route of BAPMA.

The synthetic route of BAPMA is shown in Scheme 2 according to the reported procedures [48]. 3-(BOC-amino)-1-propanol (1.47 g, 8.4 mmol), dry tetrahydrofuran (THF, 40 mL) and triethylamine (Et_3_N, 2.44 mL, 17.6 mmol) were added to a 100 mL three-neck flask with a stir bar. The reaction mixture was stirred in an ice bath and methacryloyl chloride (1.47 g, 14.1 mmol), slowly being added dropwise. When the methacryloyl chloride was added completely, the ice bath was removed and left the reaction reacted at room temperature for 24 h. After that, the mixture was filtered and the filtrate was concentrated by rotary evaporation. The crude product was washed with water 3–5 times, then dried with anhydrous sodium sulfate overnight. The oily product BAPMA was obtained through column chromatography (eluent: *V_n_*_-hexane_:*V*_ethyl acetate_ = 9:1) with a yield of 67.0%; ^1^H NMR (300 MHz, CDCl_3_, δ, ppm): δ 6.12 (s, 1H), δ 5.58 (s, 1H), δ 4.74 (s, 1H), δ 4.23 (t, 2H), δ 3.23 (q, 2H), δ 1.96 (s, 2H), δ 1.91~1.80 (m, 2H), δ 1.45 (s, 9H).

### 2.3. Synthesis of (Z)-5-(5-(4-carboxypiperidin-1-ium-1-ylidene)thiophen-2(5H)-ylidene)-2-(5-(4-carboxypiperidin-1-yl)thiophen-2-yl)-3,4-dioxocyclopent-1-en-1-olate (NIR 800)

**Scheme 3:**
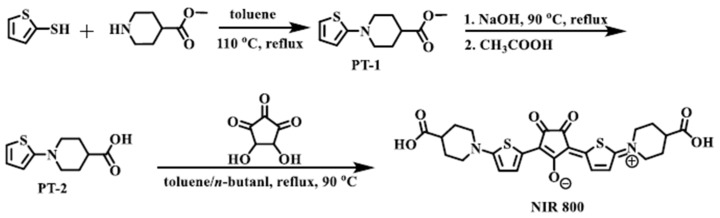
Synthetic routes of NIR 800.

The synthetic routes of NIR 800 is shown in Scheme 3 according to the reported procedures [46]. Thiophenethiol (4.87 g, 41.9 mmol) and methyl isonipecotate (9.02 g, 63.0 mmol) were added to a 100 mL three-neck flask with toluene (50 mL) and a stir bar in argon atmosphere. The solution was heated to 110 °C and reacted under reflux for 2 h. After that, toluene was removed by rotary evaporation. The solid product PT-1 was obtained through column chromatography (eluent: *V_n_*_-hexane_:*V*_ethyl acetate_ = 9:1) with a yield of 73.1%. ^1^H NMR (300 MHz, CDCl_3_, δ, ppm): δ 6.76 (d, 1H), δ 6.62 (d, 1H), δ 6.14 (d, 1H), δ 3.71 (s, 3H), δ 3.55~3.46 (m, 2H), δ 3.06~2.79 (m, 2H), δ 2.49~2.39 (m, 1H), δ 2.05~2.00 (m, 2H), δ 1.96~1.83 (m, 2H).

Secondly, PT-1 (5.02 g, 22.3 mmol) was dissolved in the aqueous solution of sodium hydroxide (0.50 mol/L), and the solution refluxed at 90 °C for 4 h. When the reaction solution was cooled to room temperature, the aqueous solution of acetic acid (1.7 mmol/L, 7.00 g of CH_3_COOH was dissolved in 70 mL of H_2_O) was added dropwise into the solution until the white precipitate appeared. Excessive acetic acid and other water-soluble impurities were removed through filtration and multiple water washings. The product was dried in a 35 °C vacuum oven overnight to obtain a light blue solid product PT-2 with a yield of 59.9%. ^1^H NMR (300 MHz, DMSO-*d*_6_, δ, ppm): δ 6.73 (d, 1H), δ 6.70 (d, 1H), δ 6.13 (d, 1H), δ 3.45~3.38 (m, 2H), δ 2.82~2.73 (m, 2H), δ 2.42~2.33 (m, 1H), δ 1.93~1.88 (m, 2H), δ 1.73~1.59 (m, 2H).

Finally, PT-2 (1.95 g, 9.2 mmol) and ketoacid (0.65 g, 4.6 mmol) were added to the mixed solvent of toluene and *n*-butanol (*V*_toluene_:*V_n_*_-butanol_ = 50 mL:50 mL) in the argon atmosphere, and refluxed at 90 °C for 2 h. The mixed solvent was removed by filtration and the soluble impurities were removed by washing with anhydrous methanol. The product was dried in a 35 °C vacuum oven overnight to obtain solid powder product NIR 800 with a yield of 84.3%. ^1^H NMR (300 MHz, DMSO-*d*_6_): δ 12.44 (d, 2H), δ 8.54 (d, 2H), δ 7.05 (d, 2H), δ 4.03~3.99 (m, 4H), δ 3.51~3.53 (m, 2H), δ 3.33~3.31 (m, 4H), δ 2.27~2.03 (m, 4H), δ 1.75~1.72 (m, 4H).

### 2.4. General Procedure for RAFT Polymerization of BnMA

A typical polymerization procedure with a molar ratio of [BnMA]_0_:[CPDN]_0_:[AIBN]_0_ = 5:1:0.3 is shown as follows: BnMA (0.50 mL, 3.0 mmol), CPDN (0.16 g, 0.6 mmol), AIBN (29.6 mg, 0.18 mmol), and toluene (1.0 mL) were added to a 5 mL ampoule with a stir bar. The ampoule was degassed by three freeze–pump–thaw cycles, purged with argon, then flame-sealed. After the desired polymerization time at 80 °C, the ampoule was taken out and cooled with ice water. THF was added to the ampoule to dissolve the polymer, and the mixed solution was precipitated in a large amount of petroleum ether and filtered. The polymer was obtained by drying under vacuum until constant weight at 35 °C. The conversion of monomer was determined gravimetrically.

### 2.5. General Procedure for Synthesis of PBnMA-b-P(BAPMA-co-PEGMA)

A typical polymerization procedure with a molar ratio of [BAPMA]_0_:[PEGMA]_0_:[PBnMA]_0_: [AIBN]_0_ = 10:10:1:0.3 is shown as follows: BAPMA (0.10 g, 0.42 mmol), PEGMA (0.21 g, 0.42 mmol), PBnMA (0.10 g, 0.042 mmol, *M*_n, GPC_ = 2400 g/mol), AIBN (2.1 mg, 0.013 mmol), and toluene (3.0 mL) were added into a 5 mL ampoule with a stir bar. All the remaining procedures were the same as the synthesis of PBnMA.

### 2.6. General Procedure for Synthesis of PBnMA-b-P(APMA-co-PEGMA)

The deprotection of PBAPMA (poly(3-(BOC amino) propyl methacrylate)) with TFA is shown as follows: PBnMA-*b*-P(BAPMA-*co*-PEGMA) (500 mg, 0.051 mmol, *M*_n, GPC_ = 9900 g/mol) was dissolved in dichloromethane (2.0 mL), and TFA (0.5 mL) was quickly added into the solution. After reacting at room temperature overnight under sealed condition, the solution was precipitated with petroleum ether twice and filtered. The block copolymer PBnMA-*b*-P(APMA-*co*-PEGMA) were obtained by drying under vacuum until constant weight at 35 °C.

### 2.7. Measurement of Critical Micelle Concentration (CMC) of PBnMA-b-P(APMA-co-PEGMA)

Pyrene (1.0 mg, 0.005 mmol) was dissolved in 5.0 mL of THF to get the solution with a concentration of 0.2 mg/mL. The pyrene solution (20 uL) was transferred into 8 clean ampoules using micropipette separately. Then the ampoules were placed in the fume hood until the THF completely evaporated. Polymer solutions at concentration ranging from 10^−7^ mol/L to 1.0 mol/L were added to the ampoules, and ensured that the concentration of pyrene was 3.0 × 10^−5^ mol/L in each ampoule. The mixed solution was stirred at room temperature for 24 h. Then measured the fluorescence intensities of the solution at 373 nm and 384 nm by fluorescence spectrophotometer (Horiba-FluoroMax-4, Hitachi High-Tech Science, Tokyo, Japan). The excitation wavelength was set to 335 nm and the slit width was 5 nm. The curve with the data of the logarithm of the concentration of polymer aqueous solutions (lg *C*) and *I*_3_/*I*_1_ (*I*_3_: fluorescence intensity of the solution at 384 nm, *I*_1_: fluorescence intensity of the solution at 373 nm) was drawn. The concentration of the polymer solution corresponding to the abrupt change point of the curve is the CMC of the copolymer.

### 2.8. Post-Modification of PBnMA-b-P(APMA-co-PEGMA) with NIR 800

Deprotected block copolymer PBnMA-*b*-P(APMA-*co*-PEGMA) (50 mg) and NIR 800 (15 mg) were dissolved in 5.0 mL dichloromethane, then the reaction solution was reacted for 24 h at room temperature. After that, the solution was precipitated in a large amount of petroleum ether. The post-modified copolymer PBnMA-*b*-P(APMA-*co*-PEGMA)@NIR 800 was obtained by suction filtration and drying in the 35 °C vacuum oven overnight.

### 2.9. Self-Assembly of Block Copolymer and Post-Modified Block Copolymer

Block copolymer (2.0 mg) was dissolved in 1.0 mL of DMF, and the solution was stirred for 1 h. Then 10 mL of deionized water was added into the solution dropwise within 2 h by a syringe pump at 25 °C. The mixed solution was placed in a dialysis bag (with a cut-off molecular weight of 3500 g/mol) and dialyzed in deionized water for 24 h by changing the deionized water every 4 h until DMF was totally removed from the solution. Then we obtained the aqueous solution of micelles. The post-modified micelles were made in the same way from post-modified copolymer PBnMA-*b*-P(APMA-*co*-PEGMA)@NIR 800. During the dialysis, the unreacted NIR 800 precipitated from the solution and can be removed by the centrifuge with a rate of 10000 r/min for 10 min.

### 2.10. Measurement of NIR Photothermal Conversion Performance of NIR 800 and Post-Modified Micelles

The NIR photothermal conversion performance of NIR 800 was represented by its temperature rising curve under NIR light. Under irradiation with NIR light (*λ*_max_ = 810 nm, 0.028 W/cm^2^), the temperatures of NIR 800 solutions at different concentrations (dissolved in DMF/pH = 8.0 buffer solutions) were measured by NIR thermal imaging camera (HT-18) at regular intervals. The temperature rising curve was made with the data of the cumulative irradiation time and the real-time temperature of the solution. The measurement of post-modified micelles was the same as the above process.

### 2.11. Experiment of Cytotoxicity

The cytotoxicity of NIR 800 on human umbilical vein endothelial cells was tested through CCK-8 (dojindo) experiment. Firstly, the human umbilical vein endothelial cells were cultured in a cell incubator at 37 °C and 5% CO_2_ for 18 h. These cells were cultured in 96-well plates (2000 per well) using endothelial cell basal medium (DMEM). After the first stage of culture, the mediums were changed into new mediums with different NIR 800 solution at different concentrations (0, 0.05, 0.25, 0.50, 1.00 mg/mL; dissolved in PBS buffer at pH = 7.4). After the second stage of culture for 48 h, CCK-8 solution (10 μL) was added to each well. Then tested the absorbance at 450 nm after 2 h. The cell survival rate was calculated by comparing with the absorbance of the cells cultured in the blank conditions.

### 2.12. Characterizations

The number-average molecular weights (*M*_n, GPC_) and molecular weight distributions (*M*_w_/*M*_n_) of polymers were determined using a TOSOH HLC-8320 (Tosoh Corporation, Tokyo, Japan) gel permeation chromatograph (GPC) equipped with a refractive index detector (TOSOH), using TSK gel guard column Super MP-N (4.6 × 20 mm^2^) and two TSK gel Supermulti pore HZ-N (4.6 × 150 mm^2^) with measurable molecular weights ranging from 5 × 10^2^ to 5 × 10^5^ g mol^−1^. DMF + 0.01 mol L^−1^ LiBr was used as an eluent at a flow rate of 0.6 mL min^−1^ operated at 40 °C. GPC samples were injected using a TOSOH plus autosampler and calibrated with polystyrene (PS) standards purchased from TOSOH (Tosoh Corporation, Tokyo, Japan). The absorption intensity of NIR light was measured by an ultraviolet spectrophotometer (UV-2600 spectrophotometer, Shimadzu Corporation, Kyoto, Japan). The test wavelength was selected from 200 nm to 900 nm. The absorption intensity of NIR light was measured by an ultraviolet spectrophotometer (UV-2600 spectrophotometer). The test wavelength was selected from 200 nm to 900 nm. The real-time temperature of different solutions under the irradiation of NIR light (λ_max_ = 810 nm, 0.028 W/cm^2^) was measured by NIR thermal imaging camera (HT-18). The ^1^H NMR spectra of compounds and polymers were measured by nuclear magnetic resonance instrument (Bruker 300 MHz, Bruker BioSpin AG, Fallanden, Switzerland), using DMSO-*d*_6_ or CDCl_3_ as the solvent and TMS as the internal standard. The size and size PDI of micelles were measured by dynamic light scattering instrument (DLS, NanoBrook 90Plus, Brookhaven Instruments, Holtsville, NY, USA). The micelles were dispersed in water and the test temperature was 25 °C. The results were averaged over five replicates measurements. The morphology and size of the micelles were measured by transmission electron microscope (TEM, HITACHI HT7700, Hitachi High-Tech Science, Tokyo, Japan) at an acceleration voltage of 120 kV. The micelles aqueous solution was carefully dripped in the center of the carbon-coated copper mesh using a micropipette. After airing for one minute, the excess liquid was removed with filter paper. The prepared copper mesh was baked under the infrared lamp for a moment to evaporate the moisture completely.

## 3. Results and Discussion

### 3.1. ^1^H NMR of BAPMA and NIR 800

According to the ^1^H NMR spectra of BAPMA (Figure S1) and NIR 800 (Figure S2), we synthesized the aim compounds BAPMA and NIR 800. The BOC protection of BAPMA (*h* in Figure S1) can be removed with TFA, and BAPMA will change to APMA which has amino group. The amino group in APMA can improve the hydrophilicity of triblock copolymers and also can react with NIR 800. The carboxyl group in NIR 800 (*a* in Figure S2c) can undergo an amidation reaction with the mentioned amino group.

### 3.2. NIR Photothermal Conversion Performance of NIR 800

**Figure 1:**
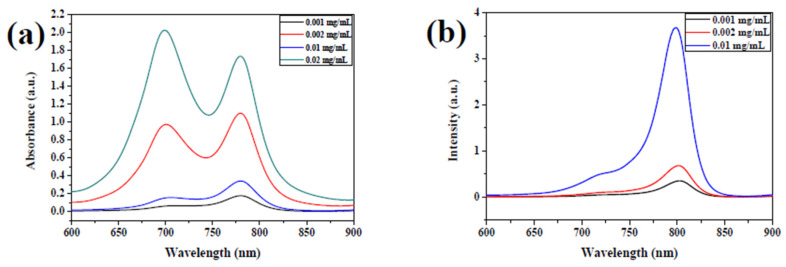
(**a**) Absorbance spectra of NIR 800 at different concentrations in buffer solution (pH = 8.0); (**b**) absorbance spectra of NIR 800 at different concentrations in DMF.

Ketocyanine dyes are well-known for their strong absorption in the NIR region [49,50]. Their photothermal conversion property is excellent, too. We synthesized symmetrical ketocyanine dye NIR 800 in this study, hoping to expand the functions of amphiphilic block copolymers with its NIR photothermal conversion property. It can be seen from the UV–vis absorption spectra in Figure 1b that NIR 800 showed a strong and sharp absorption at 800 nm. The absorption intensity gradually increased with the higher concentration of NIR 800 solution, which was dissolved in DMF or buffer solution (pH = 8.0). Because its absorption curve (shown in Figure 1a) is similar to that of post-modified micelles, the absorption value of the buffer solution at 780 nm was selected as the concentration-absorption value curve. The equation between the concentration of NIR 800 solution and absorbance at 780 nm was shown as

Y = 0.1679 + 81.285 × X(1)

(X: the concentration of NIR 800 solution; Y: the absorbance of NIR solution at 780 nm).

**Figure 2:**
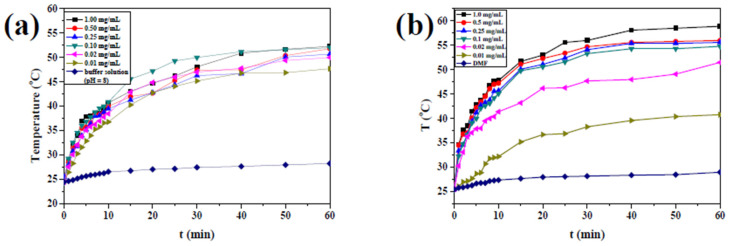
(**a**) Temperature change curves of NIR 800 at different concentrations in buffer solution (pH = 8.0) over a period of 60 min under irradiation with NIR light (*λ*_max_ = 810 nm, 0.028 W/cm^2^). (**b**) Temperature change curves of NIR 800 at different concentrations in DMF over a period of 60 min under irradiation with NIR light (*λ*_max_ = 810 nm, 0.028 W/cm^2^).

Under irradiation with NIR light (*λ*_max_ = 810 nm, 0.028 W/cm^2^) for 1 h, the increased temperature of NIR 800 solutions (dissolved in DMF or buffer solution of pH = 8.0) at different concentrations were found. When using buffer solution (pH = 8.0) as solvent, the difference in increased temperature between the solutions of different concentrations was not obvious. In Figure 2a, the temperature of solution with a concentration of 0.01 mg/mL can increase from 23 to 46 °C in 1 h, while the temperature of solution with a concentration of 1.0 mg/mL can rise from 23 to 51 °C. The difference between the highest temperatures was only 5 °C. When DMF was used as solvent, the increased temperature showed significant concentration dependence (Figure 2b). At low concentration (0.01 mg/mL), the temperature only increased by less than 15 °C in 1 h, while the increased temperature of solutions at high concentration (1.0 mg/mL) was about 35 °C. This can be explained by the specific heat capacity of different solvents. At 25 °C, the specific heat capacity of water is 4.2 kJ/ (kg × °C), and the specific heat capacity of DMF is 2.4 kJ/ (kg × K). When absorbing the same amount of heat, the solvent with larger specific heat capacity will heat up higher.

### 3.3. Synthesis and Self-Assembly of Post-Modified Copolymer PBnMA-b-P(APMA-co-PEGMA) @NIR 800

**Scheme 4:**
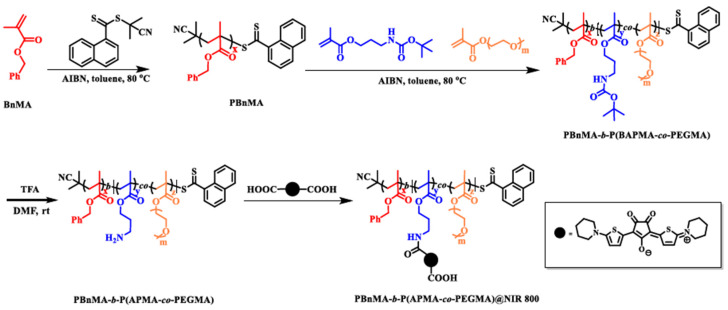
Synthetic route of PBnMA-*b*-P(APMA-*co*-PEGMA) @NIR 800. (PBnMA refers to polymers P1-P3 in Table 1. PBnMA-*b*-P(BAPMA-*co*-PEGMA) refers to block copolymers P4-P9 in Table 1. AIBN means 2,2′-azobis(2-methylpropionitrile); TFA means trifluoroacetic acid; DMF means *N*,*N*-dimethylformamide; rt means room temperature).

As shown in Scheme 4, we synthesized the block copolymer PBnMA-*b*-P(BAPMA-*co*-PEGMA) through RAFT polymerization. Firstly, we obtained the homopolymer PBnMA using CPDN as the chain transfer agent, AIBN as the initiator. The polymerization was conducted in toluene at 80 °C. Then, PBnMA was used as the macro-RAFT agent for the synthesis of block copolymer PBnMA-*b*-P(BAPMA-*co*-PEGMA). PBnMA chains formed the hydrophobic core of micelles during self-assembly; PBAPMA provided the reaction sites for amidation reaction with NIR 800 and its copolymerization with PPEGMA prevented PBAPMA being trapped in the hydrophobic core during self-assembly. After deprotection, the deprotected block copolymer experienced the post-modification with NIR 800, forming the post-modified block copolymer PBnMA-*b*-P(APMA-*co*-PEGMA)@NIR 800. NIR 800 endowed the NIR photothermal conversion property to the post-modified block copolymer. Compared with traditional physical embedding, the post-modification in the chemical method ensured the chemical combination between triblock copolymer and NIR 800, which was more favorable in the long-term cycle process. In addition, NIR 800 will absorb NIR light more fully on the hydrophilic shell, and the heating efficiency will be improved greatly.

**Figure 3:**
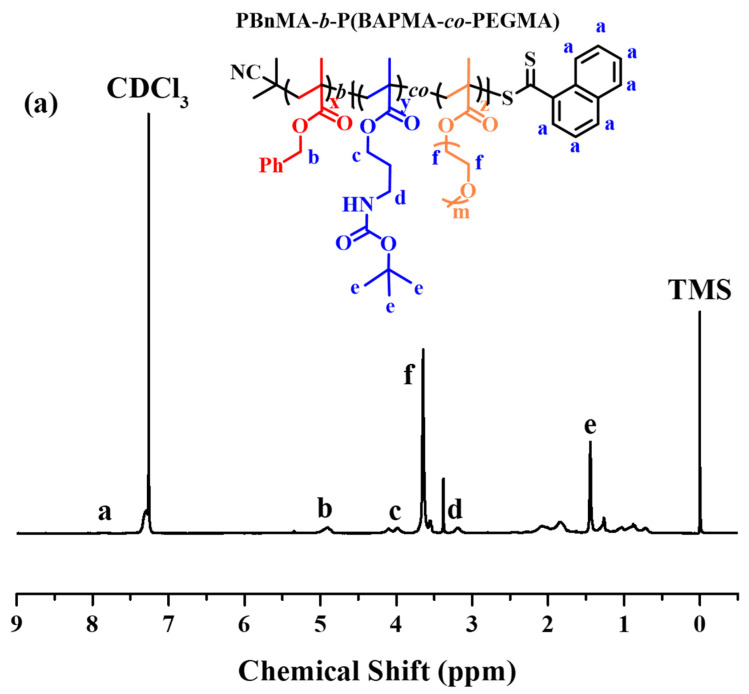

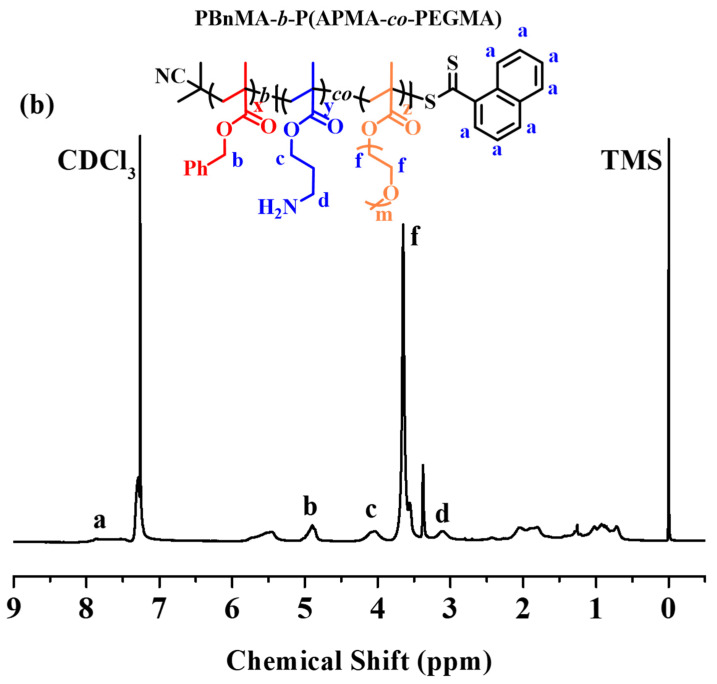
^1^H NMR spectra of (**a**) PBnMA-*b*-P(BAPMA-*co*-PEGMA) in CDCl_3_; (**b**) PBnMA-*b*-P(APMA-*co*-PEGMA) in CDCl_3_.

The size of the micelles is closely related to the molecular weights of block copolymers, as well as ratio of hydrophilic chains to hydrophobic chains. In this work, the DP of PBAPMA not only affects the size of the micelles, but also affects the efficiency of the post-modification, which is related to the NIR photothermal conversion performance. Based on the above considerations, we investigated the effect of the DP of each monomer unit on the micelle size. Long hydrophobic chains will result in larger hydrophobic core, and then the size of micelles increases. Therefore, we designed the DP of hydrophobic PBnMA at 5, 10, 15, and the DP of PBAPMA was designed at 10. In order to investigate the effect of the length of hydrophilic chains on the size of micelles, we designed the DP of PPEGMA at 5 and 10. The polymerization results of polymer PBnMA were shown in Table S1 and the polymerization results of block copolymer PBnMA-*b*-P(BAPMA-*co*-PEGMA) were shown in Table 1. As shown in Table 1, except for individual block copolymer (*M*_w_/*M*_n_ = 1.50), the molecular weight distributions were relatively narrow (*M*_w_/*M*_n_ ≤ 1.27). The corresponding GPC curves were shown in Figure S3. The structure of the block copolymer was characterized by ^1^H NMR (Figure 3a), and we can find the characteristic peaks of PBnMA at 5.12 ppm, PBAPMA at 4.20, 3.18, and 1.40 ppm, and PPEGMA at 3.50–3.70 ppm, respectively. After deprotection with TFA, the complete disappearance of the peak at 1.40 ppm was observed from ^1^H NMR spectrum (Figure 3b), which proved the removal of the *tert*-butoxycarbonyl group (BOC) and deprotected block copolymer PBnMA-*b*-P(APMA-*co*-PEGMA) was obtained.

**Figure 4:**
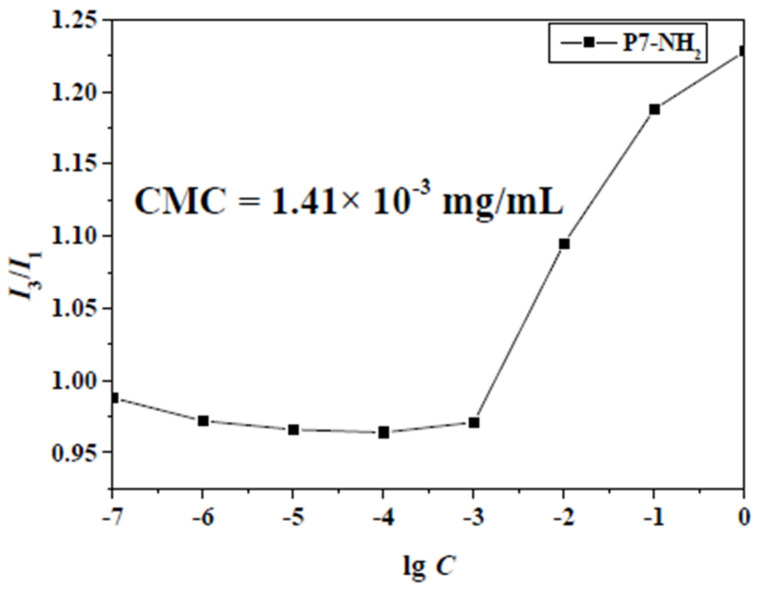
Plot of fluorescence intensity of pyrene as a function of the concentration of PBnMA-*b*-P(APMA-*co*-PEGMA) in aqueous solution. P7: PBnMA_12_-*b*-P(BAPMA_8_-*co*-PEGMA_6_). *I*_3_/*I*_1_ = A_384 nm_/A_373 nm_.

In aqueous solutions, pyrene is usually used as fluorescent probe to detect the CMC of the solutions [51]. Pyrene is nearly insoluble in water, and its *I*_1_/*I*_3_ value in aqueous solution is about 1.8 [52]. When the concentration exceeding the CMC, there will be a sudden change in the solubilizing ability of the tested solution. Therefore, the CMC can be measured by the mutation of the *I*_1_/*I*_3_ value. We used pyrene for the CMC measurement of PBnMA-*b*-P(APMA-*co*-PEGMA). In Figure 4, the curve of *I*_3_/*I*_1_ with the concentration was stable when the concentration was at 10^−7^–10^−3^ mg/mL. When the concentration was higher than 1.41 × 10^−3^ mg/mL, the value of *I*_3_/*I*_1_ increased rapidly. Therefore, the CMC of deprotected triblock copolymer P7-NH_2_ was 1.41 × 10^−3^ mg/mL.

The results of self-assembly of different block copolymers were shown in Table 2. The length of PPEGMA greatly influenced the size of micelles. With the same DP of PBnMA and PBAPMA, block copolymers with longer PPEGMA chains formed bigger micelles. For example, the size of micelles from P4 were larger than from P7; the size of micelles from P5 were larger than from P8; the size of micelles from P6 were larger than from P9. Because of the hydrophilic and hydrogen bonding interaction between PPEGMA and H_2_O, the longer PPEGMA chains will lead to the increased repulsive force between hydrophilic shells, which resulted the large sizes. According to the results in Table 2, we saw the general decrease in the size of micelles after deprotection. When PBAPMA turn to PAPMA after deprotection, the hydrophilic of polymer chains increased because of the exposed amino group [53,54]. This promised less PAPMA chains went into the core of micelles during the self-assembly, so it led to smaller micelles.

**Figure 5:**
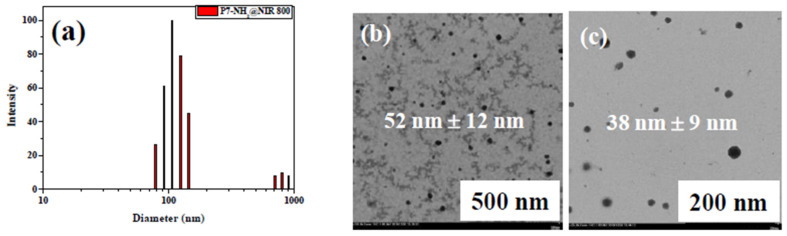
(**a**) Hydrodynamic diameters of P7-NH_2_@NIR 800 micelles; TEM images of (**b**) P7 and (**c**) P7-NH_2_@NIR 800 micelles. P7: PBnMA_12_-*b*-P(BAPMA_8_-*co*-PEGMA_6_).

Usually, a relatively small size of micelles facilitates to enhance EPR effect. Compared with other samples, the micelles from P7-NH_2_ have the smallest size, so only block copolymer P7 (P7: PBnMA_12_-*b*-P(BAPMA_8_-*co*-PEGMA_6_)) was selected as an example for post-modification with NIR 800. The ^1^H NMR spectra before and after reaction with NIR 800 were shown in Figure S4. Compared with the ^1^H NMR spectrum of P7-NH_2_, the characteristic peak signal of the amino group was significantly reduced after modification by NIR 800, indicating that most of the amino groups in block copolymer chains went through the amidation reaction with NIR 800. Furthermore, the characteristic peak signal of carboxyl group in NIR 800 was also found after modification. These results indicated successful synthesis of block copolymer P7-NH_2_@NIR 800. We obtained spherical micelles from the post-modified copolymer P7-NH_2_@NIR 800 with the size of 114 nm and PDI of 0.059 (measured by DLS) through self-assembly. We speculated that during the amidation reaction, NIR 800 not only straightly bonded to the single copolymer chain, but also underwent mild cross-linking between different polymer chains, which made the structure of the micelles denser as well as the size of micelles decreasing further. The morphologies of the micelles before and after modification were observed under TEM. From the TEM images in Figure 5b,c, both of the micelles were spherical, and the diameter of the micelles decreased to 38 nm from 52 nm post-modification.

### 3.4. NIR Photothermal Conversion Performance of Post-Modified Micelles from PBnMA-b-P(APMA-co-PEGMA)@NIR 800

**Figure 6:**
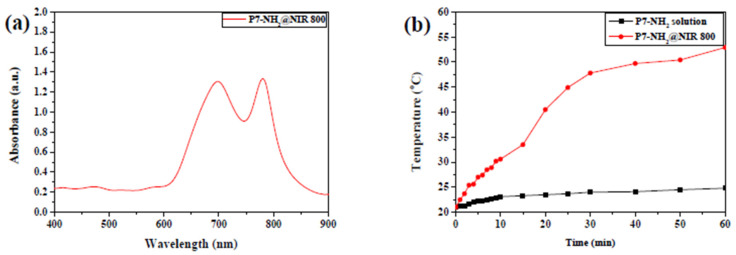
(**a**) Absorbance spectrum of P7-NH_2_@NIR 800 micelles (dilute 5 times, C_NIR 800_ = 0.07 mg/mL, bonding efficiency = 39.3%). (**b**) Temperature change curves of P7-NH_2_@NIR 800 micelles and P7-NH_2_ aqueous solution (1.0 mg/mL) over a period of 60 min under exposure to the NIR light (*λ*_max_ = 810 nm, 0.028 W/cm^2^).

Due to their suitable sizes, nanomaterials can automatically gather to target tissue. Nanomaterials used for PTT include inorganic nanoparticles, such as Au nanoparticles, cyanine dyes, as well as conjugated polymers like polypyrrole. However, inorganic nanomaterials and conjugated polymers are less soluble in aqueous solution. It hinders long-term circulation in the body. In order to solve this problem, nanomaterials made from amphiphilic block copolymers are used to load photothermal reagents. Micelles with core–shell structure are stable in body, which promises the effect of photothermal reagents. Compared with physical embedding method, chemical bond is more stable. In this work, we synthesized amphiphilic block copolymer via RAFT polymerization and subsequently modified the hydrophilic block with NIR 800 through amidation reaction, and finally obtained stable nano micelles by self-assembly with highly efficient NIR photothermal conversion property. In order to investigate the NIR light response of post-modified micelles, we measured the absorbance of micelles of P7-NH_2_@NIR 800 through UV–vis spectrum (Figure 6a). While the concentration of micelles was too high for measurement, so the aqueous solution of micelles was diluted five times. From Figure 6a, the absorbance at 780 nm of diluted solution was 1.33232 a. μ. It was calculated that the concentration of NIR 800 was 0.07 mg/mL from the concentration-absorption value curve of NIR 800. The modification efficiency (*E*_m_) was 39.3 % which was calculated from the below formula:

*E*_m_ = (*m*_detected NIR 800_/*m*_added NIR 800_) × 100% (2)

Under irradiation with NIR light (*λ*_max_ = 810 nm, 0.028 W/cm^2^), the temperature increase of deprotected block copolymer P7-NH_2_ solution was not obvious, and it just increased to 25 °C from 21 °C. Meanwhile, the temperature of micellar aqueous solution can increase from 20 °C to over 50 °C (Figure 6b) in the same conditions. The results not only confirmed the excellent NIR photothermal conversion performance of post-modified micelles, but also indicated the great potential of post-modified micelles in photothermal therapy.

**Figure 7:**
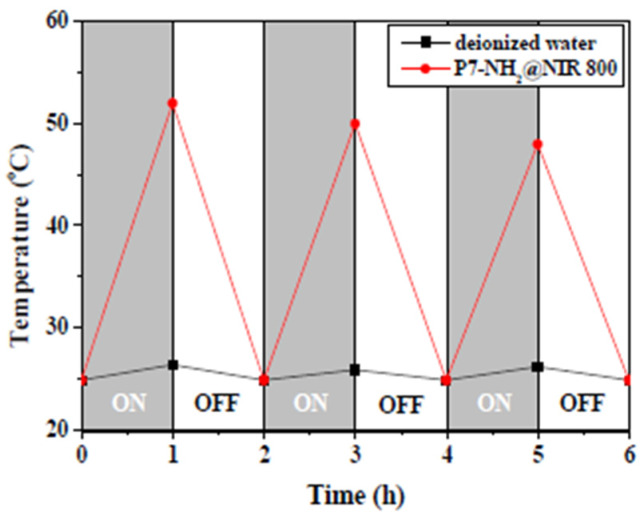
Temperature change curves of P7-NH_2_@NIR 800 micelles and deionized water under exposure to the NIR light (*λ*_max_ = 810 nm, 0.028 W/cm^2^). ‘ON’ means the NIR light is on; ‘OFF’ means the NIR light is OFF.

In order to investigate the photothermal conversion stability of the post-modified micelles from P7-NH_2_@NIR 800. The NIR photothermal conversion performance was conducted through temperature rising experiments for three cycles. The results were shown in Figure 7. When the micelle solution was under irradiation with NIR light, the temperature was increased to 52 °C in 1h, while the temperature of deionized water only increased to 26.5 °C. Then, the solution was cooled to room temperature after removing the NIR light. Experiencing three cycles like this, the temperature of post-modified micelles still could increase to 48 °C from room temperature. The results showed excellent photothermal conversion stability of the obtained post-modified micelles.

**Figure 8:**
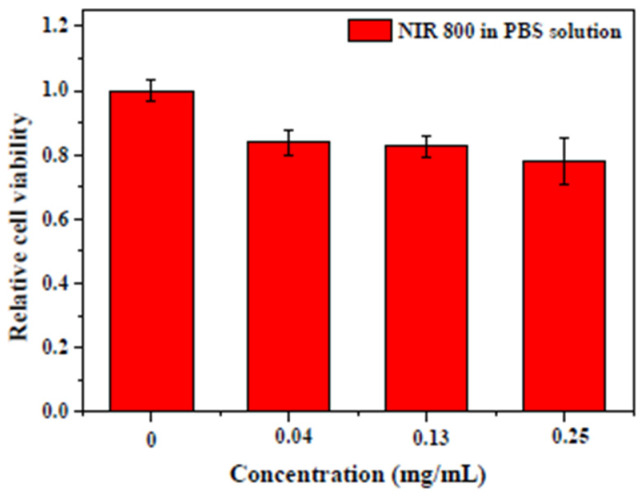
Relative cell viability of human umbilical vein endothelial incubated with a series of concentration in PBS solution of NIR 800 measured by the MTT cell viability assay.

Furthermore, the cytotoxicity of NIR 800 was evaluated through MTT assay, and NIR 800 was dissolved in PBS solution (pH = 7.4). The results of cytotoxicity experiments were shown in Figure 8. The cell viability remained at 80% after treatment with the solutions of NIR 800 at a concentration of 0.25 mg/mL or lower. The result manifested the lower toxic of NIR 800 for human vein endothelial cells, and indicated that micelles containing NIR 800 were safe enough for the potential application in vivo.

## 4. Conclusions

Herein, we successfully synthesized the functional micelles with excellent NIR photothermal conversion performance. The micelles were obtained from the self-assembly of post-modified amphiphilic block copolymer PBnMA-*b*-P(BAPMA-*co*-PEGMA)@NIR 800, which was synthesized through RAFT polymerization, deprotection, and post-modification with ketocyanine dye NIR 800. With a size of 114 nm, the micelles have potential to be used in tumor targeted therapy with the help of EPR effect. In addition, the post-modified micelles showed excellent NIR photothermal conversion property, which can be confirmed from the fact that the temperature of the modified micelles aqueous solution increased to 53 °C from 20 °C under irradiation with NIR light (λ_max_ = 810 nm, 0.028 W/cm^2^) for 1 h. Moreover, cytotoxicity experiments showed the low toxicity of NIR 800 towards human vein endothelial cells. Therefore, the post-modified micelles we synthesized have suitable size and excellent NIR light response, which provides the possibility for their potential application in tumor targeted therapy and photothermal therapy.

## Figures and Tables

**Table 1:** Molecular weight and molecular weight distribution of the resultant PBnMA-*b*-P(BAPMA-*co*-PEGMA).

Entry	R	Macro-CTA	*^a^ M*_n, GPC_ (g/mol)	*^b^ M*_n, NMR_ (g/mol)	*^a^ M*_w_/*M*_n_
**P4**	*^c^* 10/10/1/0.3	P1:PBnMA *^a^ M*_n, GPC_ = 2400 g/mol *^a^ M*_w_/*M*_n_ = 1.11 *^b^ M*_n, NMR_ = 2400 g/mol (for P4 P7)	9900	10100	1.08
**P5**			7400	10700	1.25
**P6**			6500	11200	1.5
**P7**	*^d^* 10/5/1/0.3	P2:PBnMA *^a^ M*_n, GPC_ = 3200 g/mol *^a^ M*_w_/*M*_n_ = 1.13 *^b^ M*_n, NMR_ = 3000 g/mol (for P5 P8)	6400	7300	1.14
**P8**		P3:PBnMA *^a^ M*_n, GPC_ = 3600 g/mol *^a^ M*_w_/*M*_n_ = 1.14 *^b^ M*_n, NMR_ = 3100 g/mol (for P6 P9)	5600	8800	1.27
**P9**			6900	8100	1.18

Polymerization conditions: R = [BAPMA]_0_:[PEGMA]_0_:[Macro-CTA]_0_:[AIBN]_0_, BAPMA = 0.10 g, *V*_toluene_ = 3.0 mL, T = 80 °C, time = 24 h. *^a^* Molecular weights and molecular weight distributions were determined by GPC using PS as the standard in DMF (with 0.1 wt % LiBr). *^b^* Determined by ^1^H NMR in CDCl_3_. ***^c^*** Molar ratio of P4-P6. ***^d^*** Molar ratio of P7-P9. Macro-CTA: PBnMA macro-RAFT agents with different molecular weights, P1, P2, or P3.

**Table 2:** Results of size and size PDI from the self-assembly of PBnMA-*b*-P(BAPMA-*co*-PEGMA), PBnMA-*b*-P(APMA-*co*-PEGMA), and PBnMA-*b*-P(APMA-*co*-PEGMA)@NIR 800.

Sample	*^b^* Size (nm)			*^b^* PDI		
	*^c^* P	*^c^* P-NH_2_	*^c^* P-NH_2_@NIR 800	*^c^* P	*^c^* P-NH_2_	*^c^* P-NH_2_@NIR 800
**P4: *^a^* 12-8-12**	**550**	**379**	114	0.081	0.097	0.059
**P5:***^a^* 18-7-11	337	376		0.114	0.017	
**P6:***^a^* 20-8-11	339	322		0.002	0.016	
**P7**: ^a^ 12-8-6	446	274		0.037	0.065	
**P8:***^a^* 18-9-6	324	324		0.044	0.095	
**P9:***^a^* 20-7-5	236	477		0.42	0.11	

*^a^* The actual DP of PBnMA, PBAPMA, and PPEGMA, respectively. *^b^* Size and size PDI were determined by DLS measurements. ***^c^*** P represents PBnMA-*b*-P(BAPMA-*co*-PEGMA); P-NH_2_ represents PBnMA-*b*-P(APMA-*co*-PEGMA), obtained from deprotection of *tert*-butoxycarbonyl (BOC) groups from PBnMA-*b*-P(BAPMA-*co*-PEGMA); P-NH_2_@NIR 800 represents PBnMA-*b*-P(APMA-*co*-PEGMA)@NIR 800.

## Supplementary Materials

The following are available online at https://www.mdpi.com/2073-4360/12/5/1181/s1, Figure S1: ^1^H NMR spectra of (a) PT-1 in CDCl_3_; (b) PT-2 in DMSO-*d*_6_; (c) NIR 800 in DMSO-*d*_6_. Figure S2: ^1^H NMR spectrum of BAPMA in CDCl_3_. Table S1: Molecular weight and molecular weight distribution of PBnMA macro-RFAT agent. Figure S3: GPC traces of PBnMA-*b*-P(BAPMA-*co*-PEGMA) before and after chain extension using PBnMA as the macroinitiator. Figure S4: ^1^H NMR spectra of P7-NH_2_ before (a) and after (b) with NIR 800 in DMSO-*d*_6_.
